# On improving the implementation of automatic updating of systematic reviews

**DOI:** 10.1093/jamiaopen/ooz044

**Published:** 2019-09-09

**Authors:** Anna Koroleva, Camila Olarte Parra, Patrick Paroubek

**Affiliations:** 1 LIMSI, CNRS, Université Paris-Saclay, Orsay, France; 2 Academic Medical Center, University of Amsterdam, Amsterdam, the Netherlands; 3 Department of Applied Mathematics, Computer Science and Statistics, Ghent University, Ghent, Belgium

To the Editor,

In their recent work, Martin et al.^1^ proposed an open source online tool to help update systematic reviews. The authors use a combination of machine learning and crowd-sourcing approaches to propose and assess trials that might need to be included in the update. Bibliographic databases and the ClinicalTrials.gov registry are searched for new trials to complement the updates proposed by registered users.

We believe that this work provides a very useful tool for facilitating and automating some parts of the systematic review process, which are usually time-consuming. The proposed interface is clear, user-friendly, and easy to navigate; the authors make their data freely available for registered users, so that it can be re-used in future research.

We believe that this work is of high importance, and we would like to ask for some clarifications and to provide some suggestions on how to improve the system.

## MACHINE LEARNING

The authors employ a matrix factorization approach using a shared latent space to assess the relevance of trial registry entries for each systematic review. Matrix factorization is a well-established method used in recommender systems. However, it is not commonly used for automating the screening stage of systematic reviews; in the authors’ previous work, it did not outperform the baseline approach (cosine similarity) in terms of work saved over sampling at 95% recall.

There are alternative approaches that can be more suitable for this task and could show better performance, including those using word embeddings (eg,^2^^,^^3^). In particular, Hashimoto et al.^3^ developed an approach using paragraph vectors to represent documents, as described by Le and Mikolov^4^ who proposed to map every paragraph and word to a unique vector and to further concatenate a paragraph vector with several word vectors from this paragraph to predict the next word. Paragraph and word vectors are trained using stochastic gradient descent and backpropagation. Hashimoto et al. cluster the obtained paragraph vectors by a k-means clustering algorithm to detect latent topics in the data. The final representation of documents is calculated as a k-dimensional feature vector containing distances of the given document to the k cluster centroids. The authors showed that this method outperformed the Latent Dirichlet Allocation (LDA), used by Martin et al.^1^

## CROWD-SOURCING

Crowd-sourcing can be an efficient way of collecting human annotators’ input for a particular task, but it has its drawbacks.

First of all, we would like to clarify how the quality of the contributions is controlled. The system allows only registered users to contribute, to avoid “noise” and random votes. To register, a user needs to answer some questions. However, the details on these questions and their impact on the quality control are not revealed, neither do we know whether any further requirements to qualify as a user exist. We suggest making the registration process more transparent.

One common way of crowd-sourcing quality control is to inject some gold standard data points at random intervals in the dataset and to check that they have been properly processed by the users (see eg, https://www.ucomp.eu/data/sites/16/d2.2.pdf). Of course the users do not know which of the data points are part of the gold standard dataset. This kind of quality control test could have been addressed by the authors.

Additionally, the authors do not mention if they have any system of profile management: eg, for reducing the weight of votes from a user who always votes against the majority, or for taking into account the relative experience of each user. Simple majority vote is not always the best choice to resolve disagreements, especially for a task like systematic review, where one expert vote can be more valuable than a number of nonexpert votes. Also, it is not clear if there are any strategies to tackle possible conflict of interest from the voters.

Another point to raise is the difficulty of recruiting contributors, as the authors state in their Limitations section. This is indeed a problem often undermining the development of crowd-sourcing based work, but there are successful crowd-sourcing projects in the biomedical domain, such as Cochrane Crowd (http://crowd.cochrane.org/index.html). A well-elaborated communication and dissemination plan could help tackle this issue.

## DATA

Systematic reviews are included in the system if they match two criteria: have the words “systematic review” or “meta-analysis” in the title and include at least one link to a trial in the ClinicalTrials.gov registry. The authors deliberately chose this “conservative” approach, but we would like to point out that there are alternative approaches showing operational performance, eg, the rule-based algorithm proposed by Sarker, and Diego Mollá-Aliod^5^ is reported to achieve the recall of 0.99 and precision of 1 for meta-analysis and systematic reviews. Adopting a similar approach would widen the selection of included reviews.

We would also like to raise the question of including systematic reviews that are not in open access. The researchers working on them might be interested in using the proposed tool, but it was not very clear to us from the article whether it is possible to include such a review in the system and how it is managed.

Another questionable choice is including the trial registration entries from ClinicalTrials.gov only. There is a number of other trial registries (see eg, https://www.hhs.gov/ohrp/international/clinical-trial-registries/index.html), as well as the WHO portal which provides access to a few primary registries. Including only one registry apparently limits the included trials.

The obvious reason for using ClinicalTrials.gov is the fact that metadata of articles in PubMed can contain a direct link to it, while links to other registries are not included in the metadata. However, trial registration numbers are often cited in the abstract and follow a fixed pattern including the registry name and the unique registration ID, which can be easily found with the help of regular expressions and used to automatically find the registry entry on the webpage of the corresponding registry.

## CONCLUSION

We commend the authors for their work on developing an open source online system to facilitate updating of systematic reviews. With this letter, we would like to encourage further work on this promising initiative to improve the results.

## FUNDING

This project has received funding from the European Union's Horizon 2020 research and innovation programme under the Marie Sklodowska-Curie grant agreement No 676207.

## AUTHOR CONTRIBUTIONS

AK and COP contributed to the conception of this work and wrote the first version of the manuscript. PP supervised the project and contributed to the final version of the manuscript.
